# The Effects of Orthographic Neighborhood Size and the Influence of Individual Differences in Linguistic Skills During the Recognition of Chinese Words

**DOI:** 10.3389/fpsyg.2021.727894

**Published:** 2021-11-10

**Authors:** Jianping Xiong, Yujie Zhang, Ping Ju

**Affiliations:** Department of Psychology, Faculty of Education, Henan Normal University, Xinxiang, China

**Keywords:** orthographic neighborhood size effect, Chinese words, individual differences, linguistic skills, word recognition

## Abstract

There are still inconsistencies as to whether frequency and orthographic neighborhood size affect the reading and recognition of Chinese words. In addition, research on Chinese reading still adheres to the view that “all skilled readers read in the same way” and pays little attention to the influence of individual differences in linguistic skills on word recognition. In this research, we studied the recognition of Chinese two-character words in a lexical decision task (LDT) by manipulating neighborhood size and word frequency and controlling the frequency of the initial constituent character. Individual differences in linguistic skills were assessed through tests of spelling and reading comprehension. The results showed that: (1) A larger orthographic neighborhood size of the initial character had a facilitative effect on Chinese word recognition. The orthographic neighborhood size effect is modulated by word frequency, but this modulation effect was not stable. (2) Spelling and reading comprehension skills are good indicators to assess individual differences in Chinese linguistic skills, and they are significantly correlated. (3) Individual differences in linguistic skills influence the neighborhood size effect, which is moderated by word frequency.

## Introduction

Recognizing words is the most basic and important process during reading. A large number of studies have shown that lexical properties are an important factor affecting word recognition (Wu et al., 1994; Kliegl et al., 2004; Zhao et al., 2018), and orthographic neighborhood size is one of them. The concept of orthographic Neighborhood Size (NS) was first presented by Coltheart et al. (1977) in research on word recognition in alphabetic systems. Orthographic NS is defined as the total number of words formed by replacing one letter in the target word whilst keeping other letters and positions the same. The results of research on orthographic NS in the alphabetic system (Balota and Chumbley, 1984; Andrews, 1989, 1992; Peereman and Content, 1995; Forster and Shen, 1996; Carreiras et al., 1997; Taler and Phillips, 2007; Lim, 2016) have been relatively consistent in showing a facilitative effect of NS on word recognition. That is, the more neighbor words the target word has, the more quickly and accurately it is recognized, especially for low-frequency words. The facilitative NS effect indicates that it is not only the representation of the target word that is activated but also its neighbor words during the process of word recognition.

However, due to the differences between Chinese and alphabetic systems, the conclusion that there is a facilitative NS effect in the alphabetic system is not completely applicable to Chinese. First, the number of letters composing alphabetic words is generally 2–8, and there is an inverse correlation between word length and NS such that the longer a word is, the fewer neighbor words there are (Li et al., 2015). By contrast, more than 72% of words in the Chinese system are composed of two characters (Tsang et al., 2018), making it difficult to identify a covariant relationship between word length and NS in Chinese. Therefore, the Chinese words in the present study only refer to the two-character Chinese words. Secondly, the alphabetic system is phonographic in which words are composed of a string of meaningless letters. For Chinese words, most constituent characters are not only the perceptual unit at the orthographic level but also the semantic unit at the morpheme level. Therefore, if there is an NS effect in Chinese, the mechanism of the effect could be different from that of alphabetic writing system.

### The Definition of Orthographic Neighborhood Size in Chinese Words

There are two operational definitions of orthographic NS as it applies to Chinese words. The first one, based on the definition in alphabetic system, is the total number of two-character words formed by the initial and the second character of the target word minus one (because the target word is counted twice) (Huang et al., 2006). Taking the target word “水果” as an example, there are four two-character words with “水” as the initial character, such as “水果、水稻、水面 and 水珠” (NS1 = 4), and three two-character words with “果” as the second character, such as “水果、花果 and 糖果” (NS2 = 3). Therefore, the NS of “水果” is 6 (NS1 + NS2-1 = 6). Using this definition of integrating NS is likely to confuse the different effects from initial and second constituent characters.

The second operational definition of orthographic NS is simpler in that it refers only to the initial character, not both characters, in two-character words. That is, NS is the number of two-character words with the same initial character of the target word (Tsai et al., 2006). This definition focuses on the neighbor words of initial character and prevents the confusion effects from both constituent characters. Furthermore, the regression analysis in the study of Huang et al. (2006) revealed that NS of the initial character had a greater influence on word recognition than NS of the second character. Therefore, in the current study, we also use this definition of orthographic NS, referring to the number of two-character words with the same initial character of the target word.

### Word Frequency and Orthographic Neighborhood Size

Researches on NS in the alphabetic system (Balota and Chumbley, 1984; Andrews, 1989, 1992; Peereman and Content, 1995; Forster and Shen, 1996; Grainger and Jacobs, 1996; Carreiras et al., 1997; Taler and Phillips, 2007; Lim, 2016) have been consistently revealed that the word frequency modulated the effect of NS on word recognition, specifically, the low-frequency words with more facilitative effect of NS than high-frequency words. However, in the study of Chinese words, there are still inconsistencies on whether there is an interaction between word frequency and NS.

For example, Huang et al. (2006) simultaneously manipulated NS and WF in a Lexical Decision Task (LDT) using the first definition of NS (NS1 + NS2). They found the word frequency modulated the effect of NS, which displayed that NS had a facilitative effect in recognizing high-frequency words and an inhibitory NS effect in low-frequency words. Researchers believed that the inhibitive effect of NS on low-frequency words is because most of the low-frequency words in this study have a high-frequency neighbor word, which inhibits the activation of the target word. Tsai et al. (2006) used the second definition of NS (the NS of initial character) and manipulated NS and WF to assess word recognition and sentence reading. The results showed that there was a significant interaction between WF and NS on error rate in LDT. The error rate for words with large NS was lower than those with small NS only for low-frequency words. For reaction time, although the interaction was not significant, a similar trend can be seen from the average values. For high-frequency words, the reaction time difference between words with large and small NS was 13 ms, and for low-frequency words, the difference was 20 ms. However, there was no interaction in the sentence reading task.

Thus, the interaction between WF and NS is not stable in Chinese word recognition, which may be affected by tasks and subtle changes in materials. Furthermore, neither study controlled the frequency of the initial constituent character in the two-character target words. The character frequency (CF) of Chinese words is defined as the sum of the frequencies of all words containing the same character in the same position. Therefore, the more words a character can construct, the higher frequency of this character. And it is speculated that there is a covariant relationship between the initial character frequency and the NS of Chinese two-character words. Researchers (Li et al., 2015) calculated the correlation coefficients between the number of possible word combinations by each Chinese character and its frequency in four different character databases. The results revealed the coefficients for the four databases were 0.46, 0.50, 0.62, and 0.57, respectively, furthermore, all results showed significant positive correlation (*p*s < 0.001). Therefore, the initial character frequency should be fully considered in the study of the NS effect in Chinese words (Jiang, 2006; Wu et al., 2013; Hao, 2018).

Li et al. (2015) controlled the frequency of the initial character, and then simultaneously manipulated the NS and WF. They found a significant facilitative NS effect in recognition of Chinese words, but there was no interactive effect between NS and WF in LDT (similar experimental design and results also seen in Wu et al., 2013). To explain these findings, Li et al. (2015) proposed the Semantic Activation Model for Chinese word recognition. According to this model, the recognition of Chinese words includes not only bottom-up activation from the perceptual level to the meaning level, but also top-down activation from the meaning level to the perceptual level. The perceptual level consists of character node and word node, and the meaning level consists of lemma node and semantic node. The access representations based on orthography map onto the lemma level, and in turn map onto the semantic level, which establishes an important connection between the orthographic information and higher-level semantic information. This model can explain the facilitative Chinese NS effect that was found in Wu et al.’s (2013) and Li et al.’s (2015) studies, but does not address the question of whether WF exerts an influence on the NS effect.

Combined Access Model (Caramazza et al., 1988; Schreuder and Baayen, 1997; Pollatsek and Bertram, 2000; Wang and Peng, 2000; Tse and Yap, 2018; Zhao et al., 2018) posits that morpheme features affect the recognition of high-frequency words more than low-frequency words because high-frequency words are likely stored as a whole in the mental lexicon, without activating morpheme information. Meanwhile, low-frequency words are stored as morphologically decomposed forms and activated with the morpheme. Therefore, morpheme features and word frequency could have an interaction on recognizing Chinese words. NS of a Chinese two-character word mainly embodies the word’s morpheme features. According to the Combined Access Model, this morpheme feature should also be influenced by word frequency. So, the first hypothesis in the present study is that the NS of Chinese two-character words is modulated by WF which may be manifested in the NS effect of the initial character being stronger for low-frequency words than high-frequency words. A LDT was employed in the current study in which the NS of the initial character and the WF were both manipulated, and the frequency and stroke number of the two constituent characters and the NS of the second character were strictly balanced.

### Individual Differences in Readers’ Linguistic Skills

The analysis of NS and WF effects focuses on the influence of lexical properties on word recognition. On the other hand, some researches of alphabetic languages found that individual differences in readers’ linguistic skills also affect word recognition (Kuperman and Van Dyke, 2011; Veldre and Andrews, 2015a, b; Andrews et al., 2020). For instance, compared to readers with average linguistic skills, those with high linguistic skills show shorter gaze time in recognizing targets (Haenggi and Perfetti, 1994; Ashby et al., 2005) and more automatic retrieval (Laberge and Samuels, 1974; Andrews, 2008; Kuperman and Van Dyke, 2011, 2013). They also depended less on context (Ashby et al., 2005; Andrews, 2008).

The Lexical Quality Hypothesis (LQH) (Perfetti, 2007) conceptualizes individual differences in linguistic skills in terms of the quality of lexical representations, including orthography, phonology, and meaning, in the mental lexicon. Indeed, word form knowledge (combining orthography and pronunciation) and meaning-comprehension knowledge (combining text comprehension and word meaning) have been shown to explain most of the variation in word recognition among skilled adult readers (Perfetti and Hart, 2002; Landi et al., 2006). Perfetti and Hart (2002) showed that college students with high linguistic skills had an abundance of these lexical representations, and the various types of lexical knowledge were highly integrated and tightly bound in the mental lexicon. Two factors explained these students’ word recognition: phonology and orthography. This suggests that for high-skilled readers, orthographic and phonological knowledge are integrated into forming high-quality lexical representations; orthographic information can automatically activate semantic information, and word recognition is less affected by WF and context information. By contrast, word recognition by readers with low linguistic skills is explained by three factors: phonology, orthography, and meaning-comprehension.

However, studies of Chinese reading are still based on the assumption that “all skilled readers read in the same way,” ignoring the potential influence of individual differences in linguistic skill on word recognition. In view of this fact, the second purpose of this research was to test whether individual differences in linguistic skill have an influence on Chinese words recognition.

Regarding the evaluation of individual differences in linguistic skill, Andrews (2012) pointed out that tests of reading comprehension and vocabulary can effectively assess the extraction efficiency of the lexicon and semantics, but a test of spelling skill is also necessary because spelling skill can effectively reflect lexical precision (Hersch and Andrews, 2012; Yates and Slattery, 2019). A series of eye movement studies conducted by Veldre and Andrews (2014, 2015a,b; also see Veldre et al., 2017) found that a high skipping rate is related to high spelling skill, whereas reading time is mainly related to reading skill. These findings indicate that reading comprehension and spelling skills reflect different aspects of lexical quality. In the present study, we also used a spelling test and a reading comprehension test to evaluate individual differences in linguistic skills. Our second hypothesis was that the Chinese readers’ spelling and reading comprehension skills have influence on the effect of NS and WF.

## Materials and Methods

### Participants

One hundred and twenty-one undergraduates were recruited as participants in this experiment. All participants were native Chinese speakers with normal vision or corrected-to-normal vision. They volunteered to participate in the experiment and received corresponding course credit and an appropriate reward after the experiment. The data from two participants were deleted for failing to follow the directions. The final sample size was *N* = 119 (age range = 18–23 years; 39 males).

### Materials

Based on WF and NS, 80 two-character words were selected from Chinese Linguistic Data Consortium (2003) as target words (true words), including four types: high-frequency and high NS words (HH), high-frequency and low NS words (HL), low-frequency and high NS words (LH), and low-frequency and low NS words (LL) (target words also be seen in Table A1 in Appendix). There were 20 true words of each type. As expected, there was a significant difference in WF across the four types: *F*_(3_, _76)_ = 319.52, *p* < 0.001. Multiple comparisons showed that WF did not differ for the two sets of high-frequency words (*p* = 0.79); did not differ for the two sets of low-frequency words (*p* = 0.58), but did differ for the high-frequency words and low-frequency words (*ps* < 0.001). There was also a significant difference in NS across the four types: *F*_(3_, _76)_ = 341.93, *p* < 0.001. NS did not differ for the two sets of high NS words (*p* = 0.12); did not differ for the two sets of low NS words (*p* = 0.72), but did differ for the high NS words and low NS words (*ps* < 0.001). The four types of true words were strictly matched by stroke number, character frequency, and the NS of the second character, and the differences in the four types were not significant (*ps* > 0.05) (shown in Table 1).

**TABLE 1:** Mean and standard deviation (standard deviation in brackets) of four types of true words on various indicators.

WF. condition	NS. condition	WF.(log)	1st Char.			2nd Char.		
			NS.	Freq.(log)	Stroke	NS.	Freq.(log)	Stroke
High	High	1.86 (0.35)	104.74 (31.93)	3.25 (0.24)	6.70 (2.18)	79.45 (107.90)	3.05 (0.34)	7.74 (2.93)
	Low	1.85 (0.36)	22.70 (7.53)	3.19 (0.23)	7.43 (2.17)	64.21 (37.68)	3.05 (0.38)	7.28 (2.89)
High	High	0.56 (0.41)	110.73 (34.29)	3.23 (0.23)	6.69 (2.33)	68.20 (39.35)	3.01 (0.33)	7.71 (2.81)
	Low	0.52 (0.40)	21.37 (7.82)	3.20 (0.23)	7.34 (2.00)	68.40 (45.30)	3.01 (0.34)	7.65 (2.93)
F		319.52***	341.93***	0.96	2.69	0.82	0.45	0.59

*WF.(log) means log word frequency; 1st Char. means initial characters; 2nd Char. means second characters; Freq.(log) means log frequency.*

***p < 0.001.

Eighty pseudowords were created by concatenating two characters that do not occur in the word corpus. Two characters in the pseudowords were matched with corresponding-position characters in true words on NS, frequency, and stroke number. There was no significant difference between the characters in pseudowords and the characters in true words on these variables (*ps* > 0.05). Each participant saw 160 two-character words (80 true and 80 pseudowords), which were presented randomly.

### Measures of Individual Differences in Linguistic Skills

#### Spelling Dictation Test

Spelling tests (e.g., Andrews and Hersch, 2010) often include a spelling recognition component and a spelling dictation component. Because spelling recognition is highly correlated with reading comprehension (Yates and Slattery, 2019), we assessed only spelling dictation in the current study. For this purpose, we developed a Chinese spelling dictation test based on the characteristics of Chinese. Firstly, we selected 120 low-frequency two-character words (word frequency ranged from 0.1 to 0.9 times/million) from the corpus (Chinese Linguistic Data Consortium, 2003) and constructed sentences with each word. Secondly, we recruited 50 first-year undergraduates, who did not participate in the formal experiment, to take the preliminary test. During this test, each of the target words on the spelling list was read three times. The first time it was read out in isolation; then a natural sounding sentence containing the target spelling word was read out; then the isolated word was read out again.

The participant’s task was to write down the target words they heard. An item was recorded as “correct” when both characters in each word were reproduced, and otherwise, “incorrect.” The scores on the 120 items were then analyzed using (a) biserial correlations to obtain the correlation between the accuracy on a given item (correct, incorrect) and the mean accuracy across all the items; and (b) item response theory to calculate each item’s discrimination score. Finally, thirty items that had the best scores on both indicators were selected for our formal spelling test. The average biserial correlation was 0.42 (ranging from −0.33 to 0.57 for 120 items) and an average discrimination score was 1.43 (ranging from −1.62 to 2.66 across all the items). All items in this test had factor loadings (λ) greater than 0.30, indicating an adequate prediction of the item from the latent construct assessed by the test as a whole. The Cronbach’s α = 0.93 for the spelling test in the current study. The procedure and scoring method used in the formal test were the same as in the preliminary test.

#### Reading Comprehension Test

The reading comprehension skill test was developed using eight passages from the Chinese subtest of the National College Entrance Examination from the past 10 years. Participants were asked to read the passages and answer questions about them within 30 min, at their own speed. Each passage was one paragraph long, and reading comprehension was assessed using three multiple-choice questions, each with four choices. Each correct answer was worth one point. The score on this test was the number of correct answers. The coefficient of Cronbach’s α for this test is 0.80 in the current study.

### Apparatus and Procedure

#### Apparatus

E-Prime 3.0 was used to program the LDT. The stimuli were presented on a Lenovo notebook. The resolution of the display screen was 1,280 × 720, and the distance between the display screen and the eyes of the participants was about 60 cm. The target words were presented in Song font, and the visual angle of each Chinese character was 0.6°.

#### Procedure

Each participant was given the LDT first, followed by the spelling dictation test. The reading comprehension test was administered after a 1-week interval.

LDT procedure. Participants were told that two characters would be presented on each new screen, and they should judge as quickly as possible whether the two characters together made a word. There were 5 blocks of trials. The first block included 10 practice items (5 true words and 5 false words) to familiarize the participants with the procedure. In this practice block, feedback (reaction time and accuracy) was given on each item. When accuracy on the practice items reached 90%, the participants began the LDT. The experimental materials were assigned to the subsequent four formal blocks (each block contains 20 true words and 20 pseudowords, presented randomly) and the words sharing the same characters were avoided to present in the same block following a pseudo-random design. Each trial of the LDT was as follows: (1) A fixation point “ + ” was presented in the center of the screen for 500 ms; (2) Reaction time was recorded from when the target word appeared until the participant pressed the “A” key to indicate “true word” or “L” key to indicate “false word”; (3) The blank screen was presented for 1,000 ms, then the next trial began. The experiment took about 8 min.

## Results

Linear Mixed Model (LMM) and General Liner Mixed Model (GLMM) were used for data analysis. In R 4.0.5, “lmertest” (Kuznetsova et al., 2017) package was used for model analysis, and “emmeans” 1.6.3 was used for the simple effect test. Log transforms was applied to reaction time to meet the basic assumption of the linear model.

### Neighborhood Size Effect of the Initial Character and WF Effect

The descriptive statistics for accuracy rate and reaction time under different experimental conditions are presented in Table 2.

**TABLE 2:** Accuracy rate and reaction times (mean ± standard deviation) in four conditions.

Index	H-WF		L-WF	
	H-NS	L-NS	H-NS	L-NS
ACC	0.99 ± 0.10	0.99 ± 0.12	0.88 ± 0.32	0.81 ± 0.39
RT	619.63 ± 182.78	626.63 ± 175.97	719.22 ± 225.28	744.84 ± 255.83

*N = 119. ACC means the accuracy rate under the LDT; RT means the reaction time under the LDT. H-WF means high word frequency; L-WF means low word frequency; H-NS means high neighborhood size; L-NS means low neighborhood size.*

**FIGURE 1:**
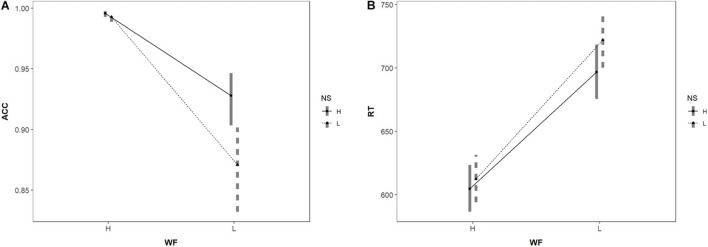
Interaction of NS and word frequency in predicting **(A)** word recognition accuracy rate, and **(B)** word recognition reaction time. WF is word frequency, which has two levels: high (H) and low (L); NS is neighborhood size: high (H) and low (L). The Error bar refers to the 95% confidence interval. Same below.

The results of the GLMM (seen in Table 3) shown that there was a significant main effect of NS on accuracy rate (*p* < 0.001); there was higher accuracy for words with many neighbors than for those with few neighbors. There was also a significant main effect of WF on accuracy rate (*p* < 0.001); there was higher accuracy for high-frequency words than low-frequency words. There was no significant interaction between NS and WF (*p* = 0.66), but exploratory analysis revealed a different NS effect for high and low-frequency words. For high-frequency words, there was a marginal significance NS effect (*p* = 0.07), while for low-frequency words, large neighborhood words had a significantly high accuracy rate than small neighborhood words (*p* < 0.001), as shown in Figure 1A.

**TABLE 3:** The results of GLMM/LMM models of neighborhood size and word frequency.

	ACC			RT		
	*b* [95%CI]	*SE*	*z*	*b* [95%CI]	*SE*	*t*
WF	−2.94	0.15	−19.53***	0.15	0.00	31.06***
	[−3.257, −2.648]			[0.015, 0.034]		
NS	−0.58	0.15	−3.89***	0.02	0.00	4.93***
	[−0.887, −0.284]			[0.143, 0.163]		
WF:NS	−0.13	0.30	−0.44	0.02	0.01	2.356*
	[−0.723, 0.482]			[0.004, 0.043]		

N = 119. *p < 0.05; ***p < 0.001.

The results of the Liner Mixed Model (LMM) (also seen in Table 3) shown that there was a significant main effect of NS on reaction time (*p* < 0.001). Reaction time was significantly shorter for words with more neighbors than words with few neighbors. There was also a significant main effect of WF (*p* < 0.001); reaction time was significantly shorter for high-frequency words than low-frequency words. The interaction effect between NS and WF was also significant (*p* = 0.02). The “simr” package 1.0.5 (Green and MacLeod, 2016) was used for statistical power test. The results showed that the statistical power of the fixed effect of interaction between NS and WF was 67.1% (95% CI = [64.09%, 70.01%]) which is higher than 50%. We can accept the result that the interaction was significant. But it also indicated that the interaction between NS and WF was not stable because the statistical power does not reach 80%. The further results of simple effects analysis showed a different NS effect on high- and low-frequency words. For high-frequency words, there was a marginal significance facilitative NS effect (*p* = 0.06), while for low-frequency words, there was a significant facilitative effect of NS (*p* < 0.05), shown in Figure 1B. That is, the NS effect was modulated by WF in Chinese two-character words recognition to a certain extent.

Some researchers (for example, Li et al., 2017) found the neighbor frequency affects the NS effect of the target word. In our target words, 65% of high-frequency words and 99% of low-frequency words contain high-frequency neighbor words, which is similar to the material in the study of Li et al. (2015). We included “whether there are high-frequency neighbor words” as a fixed factor into the original model to test the effect of this factor. The results showed that the main effect of “whether there are high-frequency neighbors” is significant (*p* < 0.001), indicating that it significantly affects the lexical decision. However, the main effect of NS and the interaction between NS and WF are still significant (*p*s < 0.05), which suggested that “whether there are high-frequency neighbors” in the current study does not affect the role of NS and WF during the word recognition.

### Analysis of Individual Differences in Linguistic Skills

Table 4 shows the descriptive statistics for the Z scores of spelling and reading comprehension tests. The normality test revealed that participants’ scores on the two tests approximated a normal distribution. There was a significant correlation between spelling dictation scores and reading comprehension scores (*r* = 0.29, *p* < 0.001), suggesting that reading comprehension skill is associated with spelling skill in Chinese reading.

**TABLE 4:** Descriptive statistics of individual difference test.

	Score range	r
ZSpelling	[−3.04 to 2.23]	0.29***
ZReading comprehension	[−1.60 to 3.45]	

N = 119. ZSpelling means the Z score of spelling test. ZComprehension means the Z score of reading Comprehension test. r means the correction between the spelling scores and reading comprehension scores. ***p < 0.001.

#### Individual Differences in Spelling Skill

**FIGURE 2:**
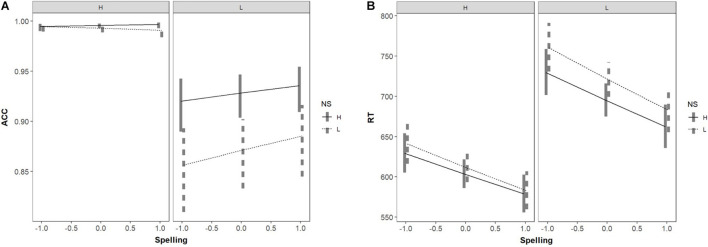
Interaction among spelling skill and NS and WF in predicting **(A)** word recognition accuracy, and **(B)** word recognition response time.

NS, WF, spelling skill, and their interaction were entered as fixed factors in GLMM to predict accuracy in word recognition. The results were shown in Table 5. There was no significant main effect of spelling skill (*p* > 0.05) and no interaction effects with other variables (*ps* > 0.05). However, considering that our research focus was on these three variables as joint influences on word recognition, we carried out further simple effects analysis of the interaction among the three factors. We divide the participants into three groups based on their spelling test scores, namely low-skilled spellers (−1), average-skilled spellers (0), and high-skilled spellers (1), the same below. Results revealed an NS effect for low-frequency words, regardless of spelling performance (*ps* < 0.05), but no NS effect on the high-skilled spellers for recognizing high-frequency words (*ps* > 0.05) (also seen in Figure 2A).

**TABLE 5:** The results of GLMM/LMM models of individual difference.

	ACC			RT		
	*b* [95%CI]	*SE*	*z*	*b* [95%CI]	*SE*	*t*
WF*NS*ZSpelling						
WF	−2.95	0.15	−19.16***	0.15	0.00	31.04***
	[−3.278, −2.655]			[0.143, 0.163]		
NS	−0.59	0.15	−3.86***	0.02	0.00	4.91***
	[−0.905, −0.287]			[0.015, 0.034]		
ZSpelling	0.05	0.09	0.60	−0.05	0.01	−3.67***
	[−0.128, 0.235]			[−0.073, −0.022]		
WF:NS	−0.11	0.30	−0.36	0.02	0.01	2.39*
	[−0.715, 0.517]			[0.004, 0.043]		
WF:ZSpelling	0.14	0.16	0.88	−0.01	0.00	−1.35
	[−0.176, 0.452]			[−0.016, 0.003]		
NS:ZSpelling	−0.24	0.16	−1.53	−0.01	0.00	−1.15
	[−0.55, 0.076]			[−0.015, 0.004]		
WF:NS:ZSpelling	0.50	0.31	1.62	0.00	0.01	0.04
	[−0.125, 1.131]			[−0.019, 0.019]		
WF*NS*ZComprehension						
WF	−2.78	0.16	−17.40***	0.15	0.00	31.09***
	[−3.094, −2.466]			[0.144, 0.163]		
NS	−0.58	0.16	−3.62***	0.02	0.00	4.96***
	[−0.894, −0.266]			[0.015, 0.034]		
ZComprehension	0.13	0.09	1.39	−0.04	0.01	−2.60*
	[−0.046, 0.306]			[−0.062, −0.087]		
WF:NS	0.02	0.32	0.07	0.02	0.01	2.40*
	[−0.607, 0.647]			[0.004, 0.043]		
WF:ZComprehension	−0.17	0.17	−1.00	0.00	0.00	−0.62
	[−0.503, 0.163]			[−0.013, 0.007]		
NS:ZComprehension	−0.27	0.17	−1.61	0.00	0.00	−0.56
	[−0.603, 0.063			[−0.012, 0.007]		
WF:NS:ZComprehension	0.74	0.34	2.20*	−0.02	0.01	−1.96*
	[0.074, 1.406]			[−0.039, 0.000]		

*N* = 119. *p < 0.05; ***p < *0.001.*

NS, WF, spelling performance, and their interactions were entered as fixed factors in the LMM to predict word recognition reaction time. The results also were shown in Table 5. The main effect of spelling score was significant (*p* < 0.001); the reaction time was significantly lower for participants with high spelling scores than for those with low spelling scores. The two-way interactions and three-way interactions were not significant (*ps* > 0.05). In order to further explore the relationship among the three variables, we made a simple effects analysis on the three-way interaction showed that NS had a facilitative effect on all spellers’ recognition of only low-frequency words (*ps* < 0.01), as was shown in Figure 2B. NS appeared to have no effect on the high-skilled and average-skilled spellers’ recognition of high-frequency words.

#### Individual Differences in Reading Comprehension Skill

**FIGURE 3:**
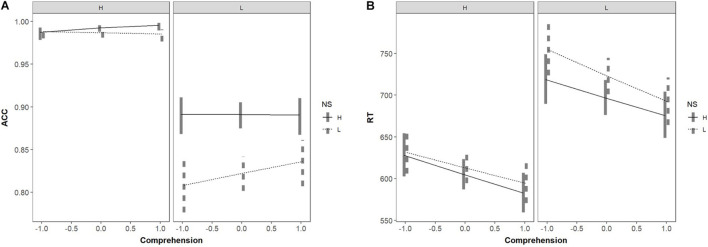
Interaction among reading comprehension, WF, and NS in predicting **(A)** word recognition accuracy, and **(B)** word recognition reaction time.

The main effect of reading comprehension was not significant in predicting word recognition accuracy (*p* = 0.17). The three-way interactions were significant (*p* < 0.05). The result of simple effects analysis was consistent with the spelling test, as is shown in Figure 3A.

The main effect of reading comprehension score in predicting reaction time was significant (*p* < 0.05). The interaction between NS, WF, and reading comprehension score was significant (*p* = 0.05). Simple effects analysis revealed a similar result with that in spelling test for low-frequency words. But, for high-frequency words, there was a significant NS effect (*p* < 0.05) for the high-skilled readers (seen in Figure 3B). As reading comprehension improved, the facilitative NS effect appeared on recognizing high-frequency words (*ps* < 0.05).

## Discussion

The NS effect has been well established in alphabetical systems, but is hard to detect in the Chinese system. In addition, research on Chinese reading still adheres to the “uniformity assumption” and pays little attention to the influence of individual differences in linguistic skills during word recognition. In the current research, we studied the NS effect and its interaction with WF in recognition of Chinese two-character words with controlling the frequency of the initial constituent character. Furthermore, we assessed the individual differences in linguistic skills through tests of spelling and reading comprehension and examined the influence of individual differences on the effect of NS and WF during word recognition.

### The Effects of Neighborhood Size and WF on Chinese Two-Character Word Recognition

The current study found a significant facilitative effect of WF, consistent with previous research (Andrews, 1989, 1992; Liu et al., 1996; Yan et al., 2006; Li et al., 2015, 2017; Sereno et al., 2020). It was also revealed that the WF effect did not vary by the NS of the initial constituent character. These results suggest that the WF effect on Chinese word recognition is stable.

Moreover, we found a facilitative NS effect on lexical recognition, consistent with previous studies on Chinese (Wu et al., 2013; Li et al., 2015) and on the alphabetic system (Andrews, 1989; Forster and Shen, 1996; Braun et al., 2006; Taler and Phillips, 2007; Carrasco-Ortiz et al., 2017). However, the mechanisms leading to similar results may be different because of the obvious contrasts between Chinese and an alphabetic writing system. An alphabetic writing system is a system of phonography, in which there is a strong connection between orthography and phonetics. The difference between neighbor words only lies in a different letter, so neighbors have very similar orthographic structures and phonetics, but no semantic correlation. Therefore, the facilitative NS effect is mainly caused by the priming effect of similar characteristics in orthography and phonetics (Newman et al., 1997; Braun et al., 2006; Carrasco-Ortiz et al., 2017).

Unlike in alphabetic systems, in the Chinese system, the target two-character word and its neighbor words share one character. Thus, the orthographic and phonetic similarity between the target word and its neighbors is only 50%, significantly lower than the similarity between neighbor words in an alphabetic system. Therefore, the facilitative effect of Chinese NS is not mainly caused by the priming effect of the orthographic and phonetic features of neighbor words, but may be related to the activation of semantic features. Because the Chinese system is ideographic, the constituent characters in compound words are similar in function to the morphemes in alphabetic words. Therefore, there is considerable semantic overlap between a Chinese word and its constituent characters (Wu et al., 2013; Li et al., 2015). Moreover, a semantic association network based on the shared initial character is formed between the target word and its neighbor words.

According to the Interactive Activation Model of Chinese word recognition proposed by Taft et al. (1999) (also see Yu and Reichle, 2017; Reichle and Yu, 2018), when a target word is recognized, it will activate its constituent characters, and the constituent characters will further activate neighbor words through serial processing. The target word is also activated bilaterally at the lexical level with words that are orthographically, phonetically, or semantically similar. Thus, the more neighbor words, the stronger the activation of target words, and the faster the response. Unfortunately, due to the limitations of the study design, we cannot directly inspect the semantic activation process of the Chinese NS effect. Future studies can inspect the Chinese NS effect by comparing performance on multiple tasks (such as naming, LDT, and sentence reading tasks) to directly verify this speculation.

We found the WF modulated the NS effect, in that the NS effect was evident primarily in recognizing low-frequency words. This result is consistent with studies on alphabetical systems (Andrews, 1989, 1992; Forster and Shen, 1996; Carreiras et al., 1997; Holcomb et al., 2002; Lim, 2016) and with our first hypothesis. The findings are in line with the AAM (Augment Addressed Morphology) model proposed by Caramazza et al. (1988). The process of lexical access includes both whole word processing and morpheme processing (Pollatsek and Bertram, 2000; Yan et al., 2006; Zhao et al., 2018); the recognition of high-frequency words is mainly based on the processing of whole words, while the recognition of low-frequency words also includes the processing of morpheme characteristics (Wang and Peng, 2000; Zhao et al., 2018). Therefore, NS and WF will interact in their effects on word recognition.

However, the effect of the interaction between NS and WS on Chinese word recognition was not stable. We speculate that neighborhood size only reflects neighbors of the initial constituent character, which is mainly related to sub-lexical features, while word frequency reflects the whole word features. The recognition of Chinese words needs to go through two steps: character processing (i.e., sub-lexical processing) and word processing. Several researchers (Li et al., 2009, 2014) have proposed that there is an interactive activation mechanism between the two processes, but due to the different time course, the interaction effect may be unstable and easily affected by experimental conditions or irrelevant variables. This is a possible reason why some studies (such as Wu et al., 2013; Li et al., 2015) failed to find this interaction effect while other studies (for example, Huang et al., 2006; Tsai et al., 2006) found the significant interaction. The Semantic Activation Model for Chinese words, proposed by Li et al. (2015), can well explain the facilitative NS effect but fails to explain the interaction between WF and NS found in this study. Therefore, future research should test the relationship between WF and NS in Chinese word recognition.

### The Influence of Individual Differences on the Effect of Neighborhood Size and WF

This study found that there was a correlation between spelling and reading comprehension, *r* = 0.29, which is consistent with the studies about the alphabetic system (Andrews et al., 2020; Yates and Slattery, 2019). Furthermore, the spelling and reading comprehension tests were similar in their association with word recognition. The higher the scores on both tests, the better the performance was on the LDT. This result can be explained by the Separate-but-Sharing Model of Jones and Rawson (2016). This model holds that spelling and reading have different cognitive mechanisms, but they share activation mechanisms through a shared response buffer. That is, spelling and reading use different orthographic representations, but both orthographic representations transfer activation to the shared response buffer, which in turn affects the units in the two orthographic representations. Thus, spelling practice will influence the orthographic representation in the spelling system, but it will also influence the orthographic representation in the reading system to a lesser degree. So, the performance in spelling test should be correlated with that in reading comprehension test.

However, compared with the results of the alphabetic system, the correlation is not high (0.29 vs. 0.54 in Yates and Slattery, 2019 and 0.39 in Andrews et al., 2020). The spelling test in the present study only focuses on spelling production, that is, spell out the corresponding words after hearing the pronunciation of words, in which the orthographic representation is activated by phonology. However, due to the large number of homonyms in Chinese, in Chinese spelling task, not only orthographic representation but also semantic representation should be activated. Therefore, in the Chinese spelling task, the activation of orthographic representation may not be as strong as that in the alphabetic system, so its influence on the orthographic representation of reading comprehension is weaker.

The results of the individual differences tests found that readers with high spelling scores had significantly higher accuracy and reaction speed in word recognition than those with low spelling scores, which is consistent with the research of Rossi et al. (2019). Burt and Tate (2002) also found that LDT performance depended on spelling accuracy in a spelling dictation task. The LQH asserts that the participants with high-quality representations can acquire lexical information more effectively than those with low-quality representations. Therefore, the difference between low- and high-skilled spellers can be reflected in the accuracy and speed of word recognition. High-quality lexical representations are more facilitative to word recognition than low-quality representations, and spelling skill can be used to measure the potential orthographic representation quality of readers. Therefore, the differences in spelling skill can be effectively reflected in the accuracy and speed of word recognition. These results also suggest that spelling skill can effectively reflect individual differences in linguistic skills in Chinese word recognition.

Furthermore, WF modulated the relationship between NS and spelling skill to some extent. Specifically, for the recognition of high-frequency words, there was an NS effect for low-skilled spellers in reaction speed but no NS effect for average-skilled spellers and high-skilled spellers. This indicated that high-frequency words are stored as a whole in the mental lexicon of average- and high-skilled spellers. Better spellers develop high-quality lexical representation, and word recognition can reach a high level of automation (Perfetti, 2007; Andrews et al., 2020), so the sub-lexical information has little impact. But for the low-skilled spellers, the high-frequency words appeared to be processed by morpheme decomposition. Therefore, they can benefit from the semantic network formed by neighbor words during recognizing high-frequency words.

For the recognition of low-frequency words, all participants showed a significant NS effect both in accuracy and reaction speed. NS affected the recognition of low-frequency words for all readers. Furthermore, with the improvement of spelling skill, the NS effect tended to be weakened gradually (spelling skill = −1 & 0, *p* < 0.0001; spelling skill = 1, *p* = 0.003), which indicated the trend that the word recognition can reach a high level of automation with the improvement of spelling skills.

Reading comprehension was also related to word recognition, which manifested that the response accuracy and speed of word recognition was significantly improved with the improvement of reading comprehension skill. This finding was consistent with studies in the alphabetic system (Veldre and Andrews, 2014; Eskenazi and Folk, 2015). Moreover, WF influenced the relationship between NS and reading comprehension skill. For low-frequency words recognition, the influence of reading comprehension skill on NS effect was similar to that of spelling skill on NS effect, which manifested those all-skilled readers can benefit from neighbor words. However, for high-frequency words, only high-skilled readers can benefit from large NS words. The reading comprehension test assesses the extraction efficiency of the lexicon and semantics. High-skilled readers can activate not only the target word but also its neighbor word because of their efficient extraction.

Our study has some limitations but also provides fruitful suggestions for future research. First, the neighbor frequency of the target word in our experimental materials was not strictly controlled. Although most of the target words have high-frequency neighbor words, this variable is not strictly balanced under the four experimental conditions. Secondly, the reliability of the reading comprehension test is not particularly high. During the process of testing, participants reported that the test was a little difficult, which may affect the reliability of this test. So, the reading comprehension test will be further revised in the future. Thirdly, the morphological family size also should be considered because there are characters that correspond to a single orthographic neighborhood but multiple morphological families. So, in the future study, we can take the morphological family size as one of the independent variables and explore the NS effect under the conditions of single or multiple morphological families, by which separating the morphological family size effect from the orthographic NS effect.

## Conclusion

In this study, we tested the influences of neighborhood size and individual differences in linguistic skills on Chinese word recognition. The following conclusions were drawn. (1) The neighborhood size of the initial character has a facilitative effect on the recognition of Chinese two-character words, and this effect is modulated by word frequency, being mainly evident in low-frequency words. However, the modulation effect was not stable and is easily affected by experimental materials and tasks. (2) Spelling and reading comprehension skills are good indicators to assess individual differences in Chinese linguistic skills, and they are significantly correlated. (3) Individual differences in linguistic skills influence the neighborhood size effect, which is moderated by word frequency.

## Data Availability Statement

The raw data supporting the conclusions of this article will be made available by the authors, without undue reservation.

## Ethics Statement

The studies involving human participants were reviewed and approved by the Henan Normal University. The patients/participants provided their written informed consent to participate in this study.

## Author Contributions

JX supervised the collection of data and wrote this manuscript. PJ and YZ collected and analyzed the data. All authors contributed to the article and approved the submitted version.

## Conflict of Interest

The authors declare that the research was conducted in the absence of any commercial or financial relationships that could be construed as a potential conflict of interest.

## Publisher’s Note

All claims expressed in this article are solely those of the authors and do not necessarily represent those of their affiliated organizations, or those of the publisher, the editors and the reviewers. Any product that may be evaluated in this article, or claim that may be made by its manufacturer, is not guaranteed or endorsed by the publisher.

## Figures and Tables

**TABLE A1:**
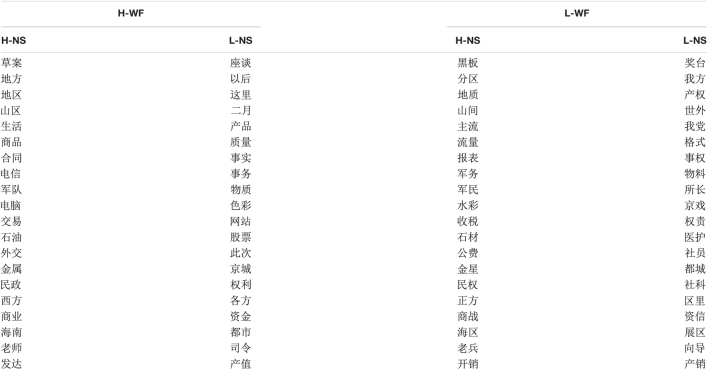
Target words under four conditions.
